# Reducing turnaround time for laboratory test results does not improve retention of stable HIV-infected adults on POV program: experience from Uganda

**DOI:** 10.7448/IAS.17.4.19607

**Published:** 2014-11-02

**Authors:** Edna Maselle, Asaph Muhanguzi, Simon Muhumuza, Jeniffer Nansubuga, Cecilia Nawavvu, Jeniffer Namusobya, Moses R Kamya, Fred C Semitala

**Affiliations:** 1 Prevention, Care and Treatment, Makerere University Joint AIDS Program (MJAP), Kampala, Uganda; 2 Monitoring and Evaluation, Makerere University Joint AIDS Program (MJAP), Kampala, Uganda; 3 Makerere University Joint AIDS Program (MJAP), Kampala, Uganda; 4 Makerere University College of Health Sciences, Medicine, Kampala, Uganda

## Abstract

**Introduction:**

HIV/ AIDS clinics in resource limited settings (RLS) face increasing numbers of patients and workforce shortage [1, 2]. To address these challenges, efficient models of care like pharmacy only visits (POV) and nurse only visits (NOV) are recommended [3]. The Makerere University Joint AIDS Program (MJAP), a PEPFAR funded program providing care to over 42,000 HIV infected adults has implemented the POV model since 2009. In this model, stable patients on antiretroviral therapy (ART) with adherence to ART >95% and Karnofsky score >90% are reviewed by a doctor every four months but visit pharmacy for ART re-fills every two months. A study conducted in August 2011 showed low retention on the POV program with symptomatic diseases, pending CD4 count, complete blood count results, and poor adherence to ART as the major reasons for the non-retention in the POV program. To improve retention on POV, the TAT (Turnaround Time) for laboratory results (the main reason for non-retention in the previous study) was reduced from one month to one week. In August 2012, the study was repeated to assess the effect of reducing TAT on improving retention one year after patients were placed on POV.

**Materials and Methods:**

A cohort analysis of data from patients in August 2011 and in August 2012 on POV was done. We compared retention of POV before and after reducing the TAT for laboratory results.

**Results:**

Retention on POV was 12.0% (95% CI 9.50–14.7) among 619 patients in 2011, (70% Females), mean age was 33 years, Standard Deviation (SD) 8.5 compared to 11.1% (95% CI 9.15–13.4) among 888 patients (70% Females), mean age 38.3 years, SD 8.9 in 2012 (p=0.59). The main reasons for non-retention on the POV program in 2012 were poor adherence to ART (23%) and missed clinic appointments (14%).

**Conclusions:**

Reducing TAT for laboratory test results did not improve retention of stable HIV-infected adults on POV in our clinic. Strategies for improving adherence to ART and keeping clinic appointments need to be employed to balance workload and management of patients without compromising quality of care, patients’ clinical, immunological and adherence outcome.
